# Gut and Vagina Microbiota Associated With Estrus Return of Weaning Sows and Its Correlation With the Changes in Serum Metabolites

**DOI:** 10.3389/fmicb.2021.690091

**Published:** 2021-08-19

**Authors:** Jia Zhang, Min Liu, Shanlin Ke, Xiaochang Huang, Shaoming Fang, Maozhang He, Hao Fu, Congying Chen, Lusheng Huang

**Affiliations:** State Key Laboratory of Pig Genetic Improvement and Production Technology, Jiangxi Agricultural University, Nanchang, China

**Keywords:** return to estrus, fecal microbiota, vaginal microbiota, sow, 16S rRNA gene sequencing, serum metabolome

## Abstract

More and more studies have indicated that gut microbiota takes part in the biosynthesis and metabolism of sex hormones. Inversely, sex hormones influence the composition of gut microbiota. However, whether microbiota in the gut and vagina is associated with estrus return of weaning sows is largely unknown. Here, using 16S rRNA gene sequencing in 158 fecal and 50 vaginal samples, we reported the shifts in the gut and vaginal microbiota between normal return and non-return sows. In fecal samples, *Lactobacillus* and S24-7 were enriched in normal return sows, while *Streptococcus luteciae*, Lachnospiraceae, *Clostridium*, and *Mogibacterium* had higher abundance in non-return sows. In vaginal swabs, the operational taxonomic units (OTUs) annotated to Clostridiales, Ruminoccaceae, and *Oscillospira* were enriched in normal return sows, while those OTUs annotated to *Campylobacter*, *Anaerococcus*, *Parvimonas*, *Finegoldia*, and *Dorea* had higher abundances in non-return sows. Co-abundance group (CAG) analysis repeated the identification of the bacterial taxa associated with the estrus return of weaning sows. The predicted functional capacities in both gut and vaginal microbiome were changed between normal return and non-return sows. Serum metabolome profiles were determined by non-targeted metabolome analysis in seven normal return and six non-return sows. The metabolite features having higher abundance in normal return sows were enriched in the pathways Steroid hormone biosynthesis, Starch and sucrose metabolism, Galactose metabolism, and Vitamin B6 metabolism, while the metabolite features belonging to organic acids and derivatives, indoles and derivatives, sulfoxides, and lignans and neolignans had significantly higher abundance in non-return sows. Correlation analysis found that the changes in gut microbiota were associated with the shifts of serum metabolites and suggested that certain bacteria might affect estrus return of weaning sow through serum metabolites. These findings may provide new insights for understanding the role of the gut and vaginal microbiota in sow return to estrus after weaning.

## Introduction

In the past decades, the reproductive performance of the pig has been improved dramatically. Weaned pigs per sow per year (PSY) in Denmark increased from 23.5 in 2000 to 33.6 in 2018 (Board, 2020). Among the reproduction traits of sows, delayed return to estrus or even non-return after weaning has been becoming one of the main problems influencing pig production. In the modern pig industry, more than 85% of sows will return to estrus within 7 days after weaning (Poleze et al., 2006; Leite et al., 2011). The interval from weaning to heat is a key indicator representing sow ability of return to reproduction after weaning and also a decisive factor affecting non-production days (NPDs) and therefore reducing PSY. The failed estrus return brings a big economic loss to the swine industry. The interval from weaning to estrus is affected by numerous factors, including nutrition, genetics, environment, management, weight loss, boar exposure, health, and mycotoxins (Levis and Hogg, 1989; Poleze et al., 2006). The heritability estimated for the interval from weaning to estrus in sows is low (0.07, 0.02, and 0.07 for the first three parities) (Leite et al., 2011), suggesting that genetics is not the major factor influencing the estrus return of weaning sows.

In recent years, more and more studies have reported the important roles of gut microbiota in pig production performances, e.g., growth, health, and even reproduction (Suo et al., 2012; Hermann-Bank et al., 2015; Ramayo-Caldas et al., 2016; Yang et al., 2018; Wang et al., 2019). Sow estrus is initiated by follicular development and the synthesis and release of sex hormones, especially estrogens. Studies have revealed that the gut microbial community plays an important role in estrogen metabolism. The use of antibiotics and the change of gut microbial community will affect host steroid hormone level in older adults (Adlercreutz et al., 1984). The decrease in the diversity of gut microbiota will impair the estrogen level (Baker et al., 2017). In humans, the systemic estrogens and estrogen metabolites are significantly affected by the composition of gut microbiota (Flores et al., 2012). In contrast, the changes of sex hormones affect the gut microbiota community in female mice (Acharya et al., 2019). Estrogen can mediate the changes of the gut microbiome in mice, which causes sex differences in obesity and metabolic syndrome (Kaliannan et al., 2018). Higher abundance of *Lactobacillus* was found in the fecal microbiota of the mice fed diet containing estrogenic isoflavones (Menon et al., 2013). To our knowledge, whether the interval from weaning to estrus is associated with the gut microbiome is largely unknown at present.

The microbiota in vagina may also relate to the reproductive performance of sows. The structure and composition of the vaginal and cervix microbiota will undergo a dramatic change in response to the pregnant condition of sows (Shuo, 2016). Miller et al. (2017) found that the bacterial composition in baboon vagina was changed profoundly under the different reproductive conditions and during ovarian cycle phases. Meanwhile, the vaginal microbiota has an important influence on human reproduction physiology including menstrual cycle (Henderson and Nibali, 2016). However, whether the vaginal microbiota is associated with the return of weaned sows is also unknown.

Therefore, in this study, we profiled the composition of gut and vaginal microbiota in more than 150 sows to analyze the relationship of the gut and vagina microbiota with sow non-return after weaning. Furthermore, we determined and compared serum metabolite profiles between normal estrus return and non-return sows to identify metabolite biomarkers associated with the failed estrus return. By integrating microbiota and metabolite data, we suggested that the changes in gut microbiota were associated with the shifts of serum metabolites and may further influence the interval from weaning to estrus.

## Materials and Methods

### Experimental Animals and Sample Collection

A total of 158 Landrace × Yorkshire F1 sows were used in this study. Most of experimental sows were at the parity 2 (58 sows), 3 (40), 4 (26), and 5 (25). The other nine sows were at the parity 6–7. All experimental sows were housed in pens with concrete slatted floor, natural light, and power ventilation, and provided corn–soybean formula diets. Sows were fed two times per day. Water was *ad libitum* available from nipple drinkers. Fecal samples were manually collected from each animal’s anus and dispensed in 2-ml tubes on the date of weaning. At the same time, a total of 50 vaginal samples were also collected from these 158 sows at the posterior region of the vagina by the sterile swabs. All samples were immediately frozen in liquid nitrogen, and stored at −80°C until use. The experimental sows had been observed estrus twice per day (8:00 a.m. and 4:00 p.m.) since their offspring were weaned at the age of 28 days. According to the interval from weaning to return to estrus, the 158 sows were classified into two groups: normal return group (144 feces samples and 39 vagina samples), sows in this group returned to estrus within 7 days after weaning; and non-return group (14 feces and 11 vaginal samples), sows in this group did not return to estrus in more than 14 days after weaning. All sows were healthy and did not receive probiotic or antibiotic therapy within 2 months of sample collection. A total of 13 blood samples were collected from the sows described above, including seven samples from normal return group, and six samples from non-return group. Serum was isolated from these 13 blood samples by centrifuging at 1500 × *g* (rcf) for 15 min and stored at −80°C until use.

### Ethics Statement

All procedures involving experimental animals satisfied the requirement of the guidelines for the care and use of experimental animals established by the Ministry of Agriculture and Rural Affairs of China. This study was approved by the Animal Care and Use Committee (ACUC) at Jiangxi Agricultural University (No. JXAU2011-006).

### Microbial DNA Extraction and 16S rRNA Gene Sequencing

Microbial DNA was extracted from feces and vaginal swab samples with the QIAamp DNA Stool Mini Kit (Qiagen, Germany) following the manufacturer’s manuals (McOrist et al., 2002). The concentration and integrity of DNA samples were measured by a Nanodrop-1000 (Thermo Fisher Scientific, United States) and 0.8% agarose gel electrophoresis. The barcode fusion primer 515F (5′-GTGCCAGCMGCCGCGGTAA-3′) and 806R (5′-GGACTACHVGGGTWTCTAAT-3′) were used to amplify the V4 hypervariable region of the 16S rRNA gene. After purification, the PCR products were used to construct the libraries and sequenced by the paired-end method on an Illumina MiSeq platform (Illumina, United States).

### Sequencing Data Analysis

Raw data of 16S rRNA gene sequencing were cleaned by filtering low-quality reads and removing the primers and barcode sequences (Fadrosh et al., 2014). Tags were assembled from high-quality paired-end clean reads by FLASH (v.1.2.11) (Magoč and Salzberg, 2011). To avoid statistical bias caused by uneven sequencing depth, the sequencing data were rarefied to 24,913 tags for each fecal sample and 27,070 tags for each vaginal sample (the lowest number of tags per sample). Operational taxonomic units (OTUs) were clustered at the cutoff of 97% sequence identity using the USEARCH (v7.0.1090) (Majaneva et al., 2015). The taxonomic assignments of OTUs were performed using the RDP classifier program (v2.2) (Wang et al., 2007). The α-diversity including observed OTUs and Chao index was analyzed by Mothur (v.1.39.5) (Schloss et al., 2009). The β-diversity was analyzed by QIIME (v.1.9.1) (Caporaso et al., 2010). A normalized OTU abundance table was used for principal coordinate analysis (PCoA) based on weighted and unweighted UniFrac distances *via* Vegan package in R (Dixon, 2003). The effect of parity on gut and vagina microbial composition of sows was also evaluated by PCoA based on weighted UniFrac distances. The PICRUSt software (v.1.0.0) was used to predict the functional capacities of the gut microbiome (KEGG Orthology) from 16S rRNA gene sequencing data against the Greengenes database (Langille et al., 2013).

Comparisons of the α- and β-diversity of the gut microbiota between normal return and non-return sows were performed using the Wilcoxon rank-sum test and PREMANOVA. Permutation was set at 10,000 times. The significance threshold was set at *p*-value < 0.05. Linear discriminant analysis effect size (LEfSe) analysis was used to identify OTUs, genus, and KEGG pathways showing differential abundances between normal return and non-return sows with the standard parameters (*p* < 0.05 and | LDA| score > 2.0) (Segata et al., 2011).

### Construction of Co-abundance Groups (CAGs) of the Gut Microbiota

Microbes that likely work together to contribute to the same ecological function could be identified by clustering co-abundance groups (CAGs) based on their co-variation of abundance (Wu et al., 2021). We constructed CAGs of the gut microbiota in experimental sows. A total of 517 OTUs that existed in at least 20% of the tested samples were used for the construction of CAGs. The correlations among 517 OTUs were calculated by the SparCC algorithm *via* the SpiecEasi package in R (Kurtz et al., 2015). Only those OTUs with SparCC correlation scores greater than 0.2 were clustered into CAGs. The correlation values were converted to a correlation distance (1–correlation value), and the OTUs were clustered using the Ward clustering algorithm *via* WGCNA package in R (Langfelder and Horvath, 2008). Similar clusters were subsequently merged if the correlation between the CAG’s eigenvectors exceeded 0.8. The CAG network was visualized in Cytoscape (v.3.7.2) (Shannon et al., 2003).

### Determination of Serum Untargeted Metabolomic Profiling and Data Analysis

Serum untargeted metabolomic analysis was performed by UPLC-QTOF-MS (ultra-performance liquid chromatography method with quadrupole time-of-flight mass spectrometry). In brief, all serum samples were thawed at 4°C and precipitated using precool methanol (Merck Corporation, Germany) at 1:3 of serum:methanol at room temperature. The mixtures were vortexed for 1 min, and then incubated at −20°C for 20 min. After centrifuged at 15,000 × *g* (rcf) for 15 min at 4°C, the supernatants were transferred into clean EP tubes and dried using a vacuum evaporator. The samples were resolved in 150 μl of water:methanol (85%:15% v/v) and stored at 4°C until measurement. A standard quality control (QC) sample was prepared by mixing and blending equal volume of each of 13 tested serum samples.

The samples were run on a 100 mm × 2.1 mm Bridged ethyl hybrid (BEH) C18 UPLC column (Waters Corporation, United States) that was packed with 1.7-μm particles by using a gradient elution of water +0.1% formic acid and acetonitrile as mobile phases. The capillary voltage was set at 3.0 kV for positive electrospray ion mode (ES+) and 2.5 kV for negative electrospray ion mode (ES−). The source and desolvation temperature were set at 120 and 350°C, respectively. Leucine enkephalin was used as the lock mass (m/z 556.2771 in ES+, and 554.2615 in ES−) at a concentration of 100 ng/ml and a flow rate of 5 μl/min for all analyses. The serum samples were eluted at a flow rate of 0.3 ml/min and a column temperature of 40°C on ES+ model for 22 min and ES− model for 18 min.

Mass spectrometry analysis was performed in both ES+ and ES− models with Waters QTOF Premier (Waters Corporation, United States). The mass range was set at 50–1200 m/z in a scan time of 0.3 s and an interscan delay of 0.02 s. System control and data collection were performed by MassLynx software (Waters Corporation, United States). The Progenesis QI software (v2.0) (Non-linear Dynamics, United Kingdom) was used for non-targeted signal detection, signal integration and feature alignment (Rusilowicz, 2016). MetaScope embedded in the Progenesis QI was used to annotate metabolites not only based on neutral mass, isotope distribution and retention time, but also based on the collisional cross-sectional area and MS/MS fragmentation data in the HMDB database. Each retained peak was then normalized to the QC sample using MetNormalize (Shen et al., 2016). The relative RSD value of the metabolites in the QC samples was set at a threshold of 30% to standardize the reproducibility of the metabolomic datasets.

Considering the limited number of metabolites (only 278 serum metabolites from pigs) in the Livestock Metabolome Database (LMDB), molecular mass data (m/z) was compared to the HMDB database (Wishart et al., 2018) to annotate the serum metabolites. If the difference between the observed mass and the theoretical mass is less than 10 ppm, the metabolite was labeled as the mass. Partial Least Squares Discriminant Analysis (PLS-DA) was performed to evaluate the differentiation of the untargeted metabolome between two groups of sows by online MetaboAnalyst (V4.0). Metabolites selected as differential candidates for further statistical analysis were identified based on variable importance in the projection (VIP) at the threshold of 1 from the PLS-DA model, which was validated at the significance threshold of *p* < 0.05, by the non-parametric univariate method (Mann–Whitney–Wilcoxon test).

### Clustering of Serum Metabolite Modules

Topological networks were constructed for serum metabolites using WGCNA in R package (Langfelder and Horvath, 2008) at a soft threshold of 9 and 5 for positive ion mode and negative ion, according to the scale-free topology criterion (*R*^2^ = 0.9). At the threshold of deepSplit of 4 and minimum cluster size of 5, metabolite modules were isolated from the topological network with the dynamic hybrid tree-cutting algorithm (Langfelder et al., 2008). The first principal component of each module was chosen as the representative measurement of its metabolic profile. Modules were subsequently merged if the correlation between the first principal components of the serum metabolite clusters exceeded 0.8. Wilcoxon rank-sum tests were performed to identify the differential metabolic modules between normal return and non-return sows at a significant threshold of *p*-value ≤ 0.05. Further pathway enrichment analysis was performed for the metabolites in differentially metabolic modules using the “untargeted metabolomic pathway analysis” function section in online MetaboAnalyst (v4.0) (Chong et al., 2019).

### Spearman Correlation Analysis Between Gut Microbiome and Serum Metabolome

A total of 13 sows (7 normal return sows and 6 non-return sows) with both serum metabolome and gut microbiome data were used to evaluate the correlation between the changes of the gut microbiome and the shifts of serum metabolome. The CAGs and metabolite modules showing differential abundances between two groups were identified by the Wilcoxon rank-sum test. Spearman correlations between CAGs and serum metabolite modules or between differential OTUs and metabolites were calculated using R software (v.3.6.1). The Benjamini–Hochberg method was used to control the false discovery rate (FDR). The visualization of the correlations was plotted using the ggplot2 package in R software (v.3.6.1).

## Results

### Comparison of Gut Microbial Composition Between Normal Return and Non-return Sows

**FIGURE 1:**
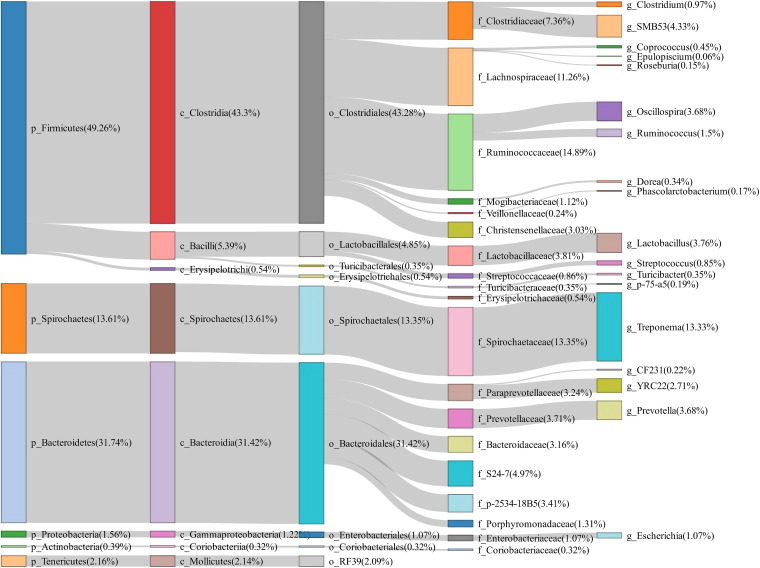
Sankey diagram showing the bacterial composition of feces samples from experimental sows. The colored columns from left to right represent the proportions of bacterial taxa from phylum to genus levels.

To evaluate the changes of fecal microbiota composition between normal return and non-return sows, a total of 158 fecal samples were collected and 16S rRNA gene sequencing was performed. The rarefaction curve suggested an adequate amount of sequencing data for the microbial diversity analysis (Supplementary Figure 1). Based on 97% similarity of the sequence identity, a total of 3,036 OTUs were obtained in fecal samples. At the phylum level, Firmicutes (49.26%), Bacteroidetes (31.74%), and Spirochaetes (13.61%) were most abundant in fecal samples. At the genus level, the relative abundances of *Treponema* (13.33%), *SMB53* (4.33%), *Lactobacillus* (3.76%), *Oscillospira* (3.68%), and *Prevotella* (3.68%) were listed in the top five (Figure 1).

We then compared the gut microbiota composition between normal return and non-return sows. Compared to normal return sows, the sows showing non-return of heat cycle after weaning had the higher observed species and Chao index, but this difference did not achieve a significant level (*p* > 0.05) (Supplementary Figure 2A). PCoA based on Weighted UniFrac distance showed a significant difference in the β-diversity of gut microbiota between normal return and non-return sows (*p* = 0.013), but not for the PCoA based on Unweighted UniFrac distances (*p* = 0.247) (Supplementary Figure 2B).

**FIGURE 2:**
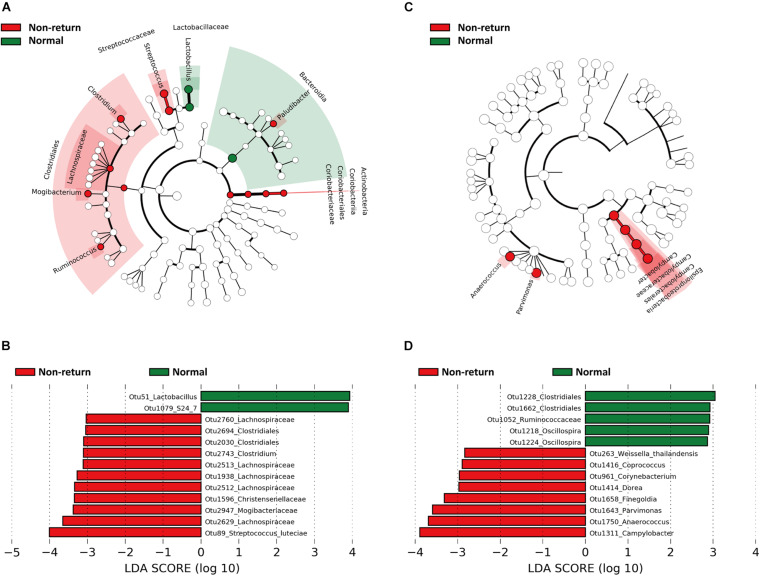
Bacterial taxa and OTUs showing different abundances between normal return and non-return sows identified by Linear discriminant analysis effect size (LEfSe) analysis. The cycles from inside to outside represent kingdom, phylum, class, order, family, and genus. **(A)** Bacterial taxa showing different abundances between normal return and non-return sows in fecal samples (| LDA| score > 2, *p* < 0.05). **(B)** Differential OTUs in fecal samples (| LDA| score > 3, *p* < 0.05). **(C)** Differential bacterial taxa in vaginal samples (| LDA| score > 2, *p* < 0.05). **(D)** Differential OTUs in vaginal samples (| LDA| score > 2, *p* < 0.05).

Linear discriminant analysis effect size was used to identify gut bacterial taxa showing different abundances between normal return and non-return sows. At the taxonomy level, we identified that Bacteroidia, Lactobacillaceae, and *Lactobacillus* were enriched in sows showing normal return, while Actinobacteria, Clostridiales, Lachnospiraceae, Streptococcaceae, *Streptococcus*, *Clostridium*, *Mogibacterium*, *Ruminococcus*, and *Paludibacter* had higher abundances in non-return sows (Figure 2A). At the OTU level, we identified a total of 55 OTUs showing different abundances between normal return and non-return sows at the significance thresholds of *p* < 0.05 and LDA > 2, including 13 OTUs with LDA > 3 (Supplementary Table 1). Among these 13 OTUs, two OTUs were annotated to *Lactobacillus* and S24-7 (Bacteroidetes), respectively, and enriched in normal return sows, and the other 11 OTUs were annotated to Mogibacteriaceae, Lachnospiraceae, Christensenellaceae, *Clostridium*, and *Streptococcus luteciae*, and enriched in non-return sows (Figure 2B).

### The Composition of Vagina Microbiota and Identification of Bacterial Taxa Associated With Non-return of Estrus in Weaned Sows

**FIGURE 3:**
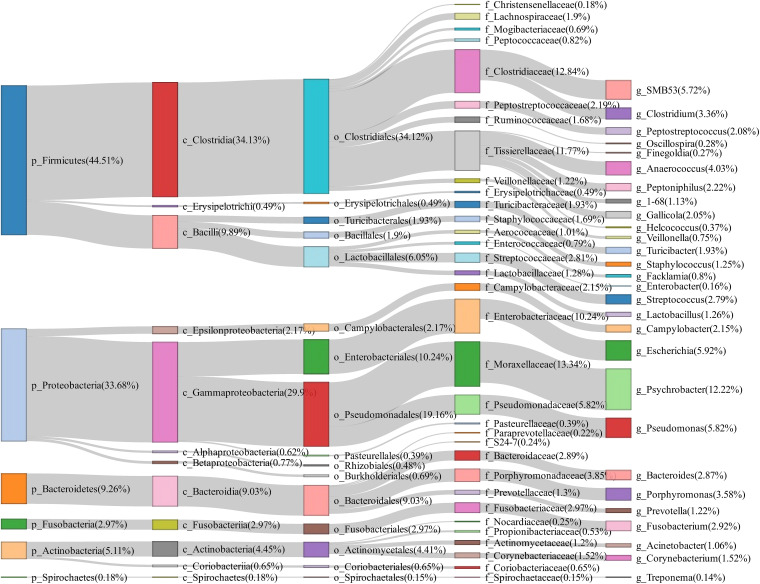
Sankey diagram showing the bacterial composition of vagina swab samples from experimental sows. The colored columns from left to right represent the proportions of bacterial taxa from phylum to genus levels.

A total of 50 vaginal swab samples were collected and 16S rRNA gene sequencing was performed. We obtained a total of 2,170 OTUs in these samples. Firmicutes (44.51%), Proteobacteria (33.68%), and Bacteroidetes (9.26%) had the highest abundance in the vagina of tested sows, and *Psychrobacter* (12.22%), *Escherichia* (5.92%), *Pseudomonas* (5.82%), *SMB53* (5.72%), and *Anaerococcus* (4.03%) were the most abundant bacterial genera (Figure 3).

We did not observe a significant difference of the α-diversity of vaginal microbial composition between normal return and non-return sows (*p* > 0.05) (Supplementary Figure 3A). PCoA based on both Weighted UniFrac distance and Unweighted UniFrac distances showed a significant difference in the β-diversity of vaginal microbiota between two groups of sows (*p* = 0.02 and 0.03) (Supplementary Figure 3B).

The bacterial taxa showing different abundances in vaginal samples between normal return and non-return sows were identified by LEfSe. Epsilonproteobacteria (including campypylobacterales, campylobacteraceae, and *Campylobacter*), *Anaerococcus*, and *Parvimonas* had higher abundances in non-return sows (Figure 2C). At the OTU level, a total of 13 OTUs showed different abundances between normal return and non-return sows (*p* < 0.05 and LDA > 2). Among them, five OTUs annotated to Clostridiales, Ruminoccaceae, and *Oscillospira* were enriched in normal return sows, while those OTUs annotated to *Campylobacter*, *Anaerococcus*, *Parvimonas*, *Finegoldia*, and *Dorea* had higher abundances in non-return sows (Figure 2D).

Some bacterial taxa showing different abundances between normal return and non-return sows were identified in both feces and vagina samples. For example, *Oscillospira* and Ruminococcaceae were enriched in normal return sows, while *Coprococcus*, Lachnospiraceae, and *Dorea* were enriched in non-return sows (Supplementary Table 1 and Figure 2D).

### CAGs of OTUs Associated With the Return of Estrus in Weaned Sows

**FIGURE 4:**
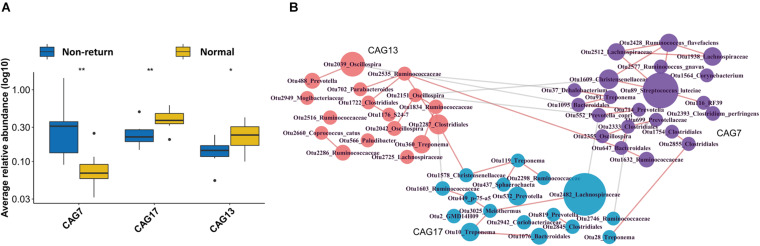
Identification of differential Co-abundance group (CAGs) between normal return and non-return sows. **(A)** Three CAGs showing different enrichments between normal return and non-return sows. Wilcoxon rank-sum test was used to test the different enrichments, *p ≤ 0.1, **p ≤ 0.05. **(B)** The horizontal network diagram of three CAGs showing different abundances between normal return and non-return sows. The diagram shows that the OTUs were enriched between normal return and non-return sows. The name of each OTU node was annotated based on the RDP classifier and the size of the nodes represent the abundance of OTU. The thickness of the connections between nodes indicates the weight of the correlation coefficient between OTUs. Lines were drawn only when its correlation coefficient is greater than 0.2. The red lines represent a positive correlation and the gray lines represent a negative correlation. The colors of the nodes represent different types of CAG.

As a complex microecological system, gut microbes interact with each other and form functional groups. A co-abundance network of OTUs was constructed in 13 samples from the sows, which also had serum metabolomic data (to further analyze the correlation between CAGs and metabolite modules). The 517 OTUs present in at least 20% of the samples were co-clustered based on the SparCC correlation coefficient. A total of 30 CAGs were obtained (Supplementary Table 2). Among them, CAG7 and CAG17 showed significantly different enrichments between normal return and non-return sows (Figure 4A, *p* = 0.01 and 0.05, respectively), and CAG13 showed the tendency of association with normal return of estrus (Figure 4A, *p* = 0.1). CAG13, which included the OTUs that were annotated to *Oscillospira*, Ruminococcaceae, Clostridiales, and *Parabacteroides*, and the CAG 17, which was composed of the OTUs that were annotated to Lachnospiraceae, *Treponema*, and Bacteroidales, were significantly enriched in normal return sows. Conversely, CAG7, which was composed of 21 OTUs, including OTU 89 (*S. luteciae*), OTU 2428 (*Ruminococcus flavefaciens*), OTU 2577 (*Ruminococcus gnavus*), OTU 2512 (*Lachnospiraceae*), OTU 1928 (*Lachnospiraceae*), OTU 552 (*Prevotella copri*), and OTU 2393 (*Clostridium perfringens*), was significantly enriched in non-return sows (Figure 4B). Particularly, OTU 89 (*S. luteciae*) was the hub OTU in the CAG7 and also identified to enrich in non-return sows at the OTU level (Supplementary Figure 4).

### The Changes of Potential Functional Capacity of Fecal Microbiome and Vaginal Microbiome Between Normal Return and Non-return Sows

**FIGURE 5:**
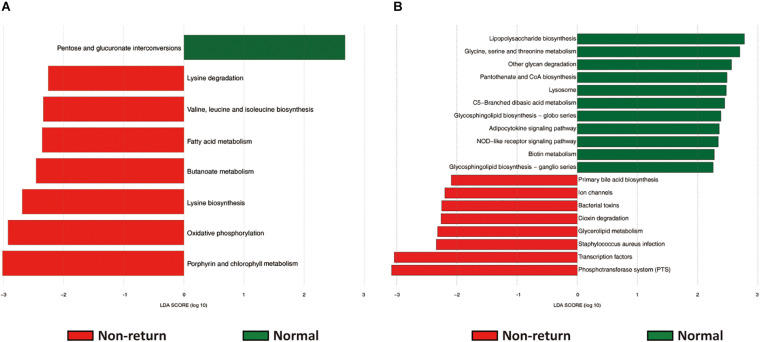
The predicted function capacities of the gut and vaginal microbiome showing different abundances between normal return and non-return sows. Linear discriminant analysis effect size (LEfSe) analysis was used to identify differential KEGG pathways (level 3). **(A)** Differential KEGG pathways identified in fecal samples (| LDA| score > 2, *p* < 0.05). **(B)** Differential KEGG pathways identified in vaginal samples (| LDA| score > 2, *p* < 0.05).

The potential functional capacities of both fecal and vaginal microbiome were predicted by PICRUSt based on 16S rRNA gene sequencing data. We investigated the shifts of potential functional capacities of gut and vaginal microbiome between normal return and non-return sows. At level 3 of KEGG pathways, we identified a total of eight pathways in the fecal microbiome showing differential abundances between two groups of sows. Among them, seven pathways had higher relative abundances in non-return sows, namely, Lysine biosynthesis; Fatty acid metabolism; Valine, leucine, and isoleucine biosynthesis; Lysine degradation; Oxidative phosphorylation; Butanoate metabolism; and Porphyrin and chlorophyll metabolism (LDA score > 2), while only one pathway (Pentose and glucuronate interconversions) was significantly enriched in normal return sows (Figure 5A). In the vaginal microbiome, 19 KEGG pathways had different enrichments between two groups of sows, namely, 11 pathways significantly enriched in normal return sows, such as Pantothenate and CoA biosynthesis, Metabolism of cofactors and vitamins; Glycine, serine, and threonine metabolism; NOD-like receptor signaling pathway; and Other glycan degradation, and 8 pathways enriched in non-return sows, including Bacterial toxins, *Staphylococcus aureus* infection, Glycerolipid metabolism, and Dioxin degradation (Figure 5B and Supplementary Table 3).

### Comparison of Serum Metabolome Profiles Between Normal Return and Non-return Sows

**FIGURE 6:**
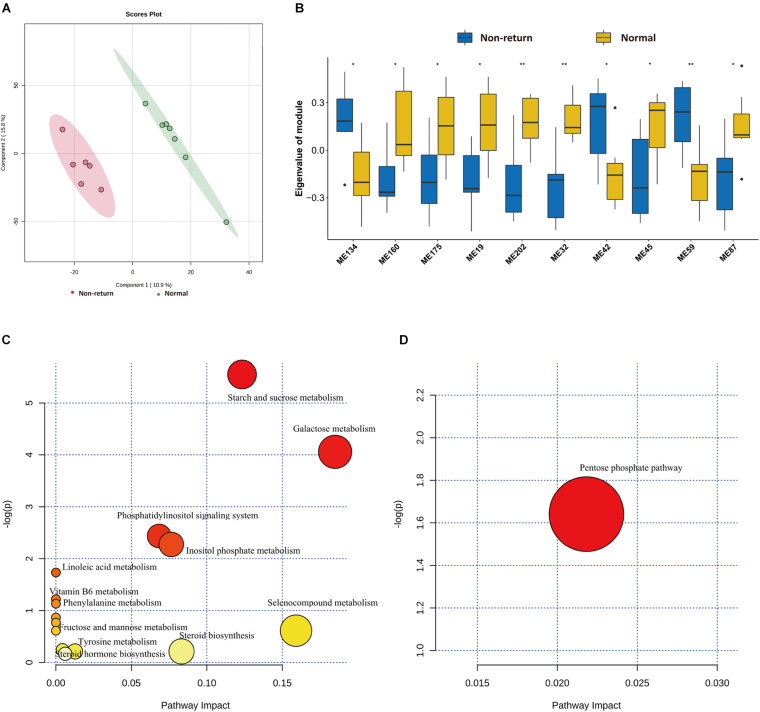
Identification of differential metabolic profiles between normal return and non-return Sows. **(A)** PLS-DA diagram of serum metabolic profiles shows significant differences of serum metabolites profiles between normal return and non-return sows. **(B)** Box plots indicate 10 co-abundance clusters of serum metabolites between normal return and non-return sows. *t*-test was used for difference analysis, * *p* < 0.05, ** *p* < 0.01. **(C)** The KEGG pathway enriched by serum metabolite features having higher abundance in serum of normal sows. **(D)** The KEGG pathways enriched by serum metabolite features having higher abundance in serum of non-return sows. The *x*-axis and the size of dots indicate the KEGG pathway impact of differential serum metabolite features, and the *y*-axis shows the –log_10_ of significant *p*-value in metabolite enrichment analysis. The size and color of dots represent the value of the KEGG pathway impact.

To evaluate the shifts of serum metabolites between normal return and non-return sows, non-targeted metabolomics analysis was performed in 13 serum samples described above (method). After quality control, a total of 3,813 metabolite features were obtained for subsequent analysis, including 2,402 metabolite features from positive ion mode and 1,411 features from negative ion mode. Significant shifts of serum metabolites were observed between normal return and non-return sows (Figure 6A). We identified a total of 32 metabolites showing different abundances between normal return and non-return sows. These metabolite features were annotated according to the HMDB database. The metabolite features enriched in normal return sows were mainly annotated to carbohydrates and carbohydrate conjugates, fatty alcohol esters, zearalenones, and hydropyridines, while those metabolite features enriched in non-return sows were annotated to diterpenoids, fatty acids and conjugates, hydroxycinnamic acids and derivatives, fatty alcohols, tropane alkaloids, indoles, and derivatives (Supplementary Table 4).

Considering the complex relationships among serum metabolites, 3,813 metabolite features in all 13 samples were clustered into 273 co-abundance modules using WGCNA, including 208 modules in positive ion mode and 65 modules in negative ion mode. Among these modules, 10 modules showed differential enrichments between normal return and non-return sows (Figure 6B). Seven metabolite modules containing 57 metabolite features were significantly enriched in normal return sows. These 57 metabolite features were annotated to organic compounds, flavonoids, lipids and lipid-like molecules, carbohydrates and carbohydrate conjugates, indolyl carboxylic acids and derivatives, and vitamin D and derivatives (Supplementary Table 5), and were enriched in the pathways Steroid hormone biosynthesis, Starch and sucrose metabolism, Galactose metabolism, and Vitamin B6 metabolism (Figure 6C). Three metabolite modules containing 23 metabolite features had significantly higher abundance in non-return sows. These 23 metabolite features belonged to tropane alkaloids, indoles and derivatives, sulfoxides, lignans, and neolignans (Supplementary Table 5), and were significantly enriched in Pentose phosphate pathway (Figure 6D).

### The Correlation Between the Changes in Gut Microbiota and the Shifts of Serum Metabolites

**FIGURE 7:**
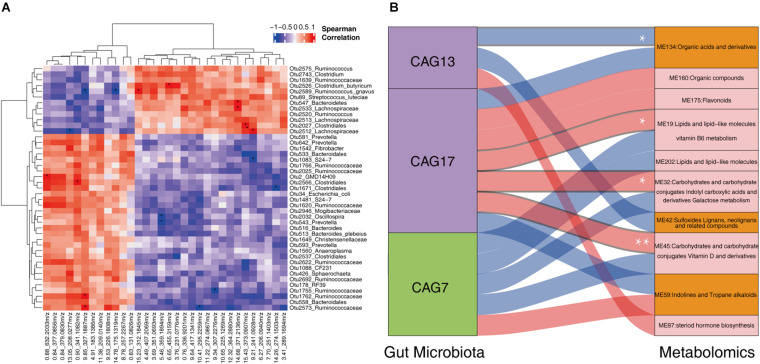
The correlations between fecal microbiota and host serum metabolite profile. **(A)** The heatmap of the correlations between differential fecal microbiota and serum metabolites. Spearman correlation coefficients were calculated. Red represents positive correlation and blue represents negative correlation. The star in the grid indicates the significant threshold *FDR < 0.05. **(B)** Spearman correlation analysis between the CAGs and the metabolite modules. The connections with a coefficient greater than 0.4 were plotted. The thickness of the connections between CAGs and metabolic modules represents the weight of the correlation coefficient. Red connections represent positive correlation, and blue connections represent negative correlation. In the column of CAGs, the green box represents the CAGs that were significantly enriched in non-return sows, and the purple boxes represent the CAGs that were significantly enriched in normal return sows. In the metabolome column, the orange boxes represent metabolic modules that were significantly enriched in non-return sows, and the pink boxes represent metabolic modules significantly enriched in normal return sows. *FDR < 0.05, **FDR < 0.01.

We further evaluated the correlation between the shifts of fecal microbiota and the changes of host serum metabolites in 13 sows described above. LEfSe analysis identified 43 OTUs showing differential abundance between normal return and non-return sows (Supplementary Figure 4, | LDA| > 2, *p* < 0.05). Most of these differential OTUs (27/43) were also identified in the whole dataset (158 samples), such as those OTUs annotated to Bacteroidales, S24-7, Ruminococcaceae, *Oscillospira*, Christensenellaceae, Sphaerochaeta, *Ruminococcus*, Clostridiales, Ruminococcaceae, Lachnospiraceae, *Clostridium butyricum*, *Clostridium*, and *S. luteciae*. We then analyzed the correlations between 43 differential OTUs and 32 differential serum metabolites described above. At the significance threshold of FDR < 0.05, we identified 20 significant correlations. OTU 2526 (*C. butyricum*), OTU 2589 (*R. gnavus*), OTU 2027 (Clostridiales), OTU 2512 (Lachnospiraceae), OTU 547 (Bacteroidetes) and OTU 2533 (Lachnospiraceae), all of which had higher abundances in the gut microbiota of non-return sows, were positively correlated with (3 beta, 17 alpha, 23S)-17,23-Epoxy-3,28,29-trihydroxy-27-norlanost-8-en-24-one (6.65_455.3159 m/z), Alosetron (15.23_312.1845 m/z), 3-Feruloyl-1,5-quinolactone (15.43_373.0907 m/z), Erythrose (9.21_241.0928 m/z), and Ecgonine (14.68_371.2136 m/z), respectively (FDR < 0.05). These five metabolites also had higher abundances in non-return sows (Supplementary Table 4). The OTU 2 (GMD14H09), OTU 2566 (Clostridiales), OTU 1762 (Ruminococcaceae), and OTU 2573 (Ruminococcaceae), which showed enrichments in normal return sows, showed positive correlations with the metabolites enriched in normal return sows [3′-Sialyllactose (0.88_632.2033 m/z), Lactulose (0.90_341.1082 m/z), and Metixene (9.85_327.1887 m/z), respectively]. We also observed the significant correlations between those differential OTUs and serum metabolites, which showed different directions of enrichment between normal return and non-return sows. For example, OTU 2589 (*Ruminococcus gnavus*) was negatively correlated with Metixene (9.85_3.27.1887 m/z) and 2,4,6-Triethyl-1,3,5-trioxane (14.78_17 5.1319 m/z) (Figure 7A).

We then evaluated the correlations between three differential CAGs (CAG7, CAG13, and CAG17) and 10 differential metabolite modules (ME32, ME19, ME42, ME134, ME160, ME175, ME202, ME45, ME59, and ME87). Those correlations with coefficient (*r*^2^) > 0.4 were selected. CAG17 was positively correlated with ME45 (mainly including Carbohydrates and carbohydrate conjugates, and Vitamin D and derivatives), ME32 (Carbohydrates and carbohydrate conjugates, and Indolyl carboxylic acids and derivatives), and ME19 (Lipids and lipid-like molecules, related to Vitamin B6 metabolism), and CAG13 was negatively correlated with ME134 (Organic acids and derivatives) (FDR < 0.05, Figure 7B). However, no significant correlation was identified between CAG7 and metabolite modules. It implies that the changes in the fecal microbiota might lead to the shift of serum metabolites and was further associated with non-return in weaned sows.

## Discussion

The interval from weaning to heat return has a significant effect on sows PSY. It affects the economic benefit of the pig industry. In this study, we explored the relationship between fecal and vaginal microbiota and the heat return of weaned sows. The serum metabolome was also measured to identify metabolites associated with non-return of estrus in weaned sows. We found that the changes in gut microbiota contributed to the shifts of serum metabolites and may further affect sow estrus return after weaning. To our knowledge, we firstly investigated the effect of gut and vaginal microbiota composition with sow estrus return of weaned sows by integrating serum metabolome analyses.

Consistent with the previous reports (Xiao et al., 2017; Dong-Jie et al., 2018), Firmicutes, Bacteroidetes, and Spirochaetes are the most abundant phyla in the fecal microbiota of sows. However, different from that in the gut, Firmicutes, Proteobacteria, and Bacteroidetes were the dominant phyla in vaginal bacterial communities, and this result was consistent with Kwawukume’s observation (Kwawukume, 2017). Recent studies have demonstrated that the prevalence and relative abundance of the genus *Lactobacillus*, which is the most abundant genus in women’s vagina and helps host against pathogens and infection (Ventolini, 2015), varied in the vagina across different mammalian species (Miller et al., 2016). In this study, *Lactobacillus* only accounts for an average of 1.31% of relative abundance in all tested vaginal samples. A previous study indicated that Lactobacillaceae had high abundances in the vaginal of Göttingen minipigs and showed no significant changes during heat cycle (Lorenzen et al., 2015).

Different parities may affect the gut microbial composition of sows (Gaukroger et al., 2021). However, in this study, we did not observe the significant difference of the microbial compositions of the gut and vagina among parities (Supplementary Figure 5). This should be due to the fact that most experimental sows were at the parity 2 to 5 (94.3%). Their ages were not significantly different. Similar to the result reported previously (Xu et al., 2020), non-return sows had higher observed species (*p* = 0.08) and Chao index (*p* < 0.05) of fecal microbiota than normal return sows. This may be due to the fact that sex hormones in normal return sows reduced the diversity of gut microbiota. We identified gut and vaginal microbes associated with the return to estrus of weaned sows. *Lactobacillus*, S27-4, and Bacteroidia were enriched in the gut of normal return sows. *Lactobacillus* was associated with low body mass index (BMI) or normal weight (Choi et al., 2019; Zhu et al., 2019). It has also been reported that both feed-induced obese Ossabaw miniature pigs and genetically obese Iberian pigs exhibit poor reproductive performance (Gonzalez-Añover et al., 2011; Newell-Fugate et al., 2014). Severely obese women show a higher probability of hormonal disorders, menstrual disorders, anovulatory cycles, and ovulation abnormalities than normal-weight women (Hartz et al., 1979). Mouse model experiments show that obesity is negatively correlated with estrus performance and leads to infertility (Bermejo-Alvarez et al., 2012). S24-7 is a family belonging to Bacteroidetes, which widely exists in the intestinal tract of animals. It is now named Muribaculaceae (Lagkouvardos et al., 2019). The abundance of S24-7 in the gut of sows is increased during pregnancy and decreased during weaning (Ji et al., 2019). The S24-7 has the capacity to degrade complex carbohydrates (Lagkouvardos et al., 2019), and members of *Bacteroides* have plant polysaccharide degrading enzymes, which can participate in polysaccharide degradation (Zou et al., 2019). CAG13 and CAG17 were enriched in normal return sows. These two CAGs were composed of OTUs belonging to the bacteria mainly related to anti-inflammatory and polysaccharide metabolism, including Ruminococcaceae, Lachnospiraceae, and *Oscillospira* (Scott et al., 2014; Konikoff and Gophna, 2016; Esquivel-Elizondo et al., 2017). Ruminococcaceae and Lachnospiraceae have been reported to produce butyrate, which plays the roles in anti-inflammatory response (Liu et al., 2019). *Oscillospira* can use the host glycogen to produce energy (Kohl et al., 2014), and the abundance of *Oscillospira* was significantly reduced in patients with inflammation (Gophna et al., 2017). Studies have shown that Ruminococcaceae, Lachnospiraceae, *Oscillospira*, and *Treponema* can ferment dietary fiber (Niu et al., 2015; Upadhyaya et al., 2016; Yu-Jiao et al., 2016). Conversely, *Streptococcus luteciae* (Liang et al., 2014), *Ruminococcus gnavus* (Hall et al., 2017), *Prevotella copri* (Pedersen et al., 2016), and *Clostridium perfringens* (Cree et al., 2016), which were annotated to the OTUs in the CAG7 have been reported to associate pro-inflammatory.

We showed that serum metabolites had a significant shift between normal return and non-return sows. This result was consistent with a previous report (Xu et al., 2020). Carbohydrates and their conjugates, and lipids and lipid-like molecules were enriched in normal return sows, Carbohydrates and lipids can compensate for the energy loss and meet the nutritional requirements during sow lactation. Generally, most sows will encounter energy loss and weight loss during lactation. After weaning, sows need to replenish energy and nutrition for estrus again. The metabolites showing different abundances between normal return and non-return sows were enriched to the pathways of galactose metabolism, starch and sucrose metabolism, and vitamin B6 metabolism. This result suggested that the differential metabolites may provide sows with the energy and vitamin requirements for estrus. The supplement of vitamin B6 can significantly increase the production of luteinizing hormone (LH) in sows during estrus (Dalto et al., 2015). Unexpectedly, the metabolites of Metixene (9.85_3.27.1887 m/z) and Astemizole (15.88_459.2560 m/z) were enriched in normal return sows. Both Metixene and Astemizole can be used to relieve or treat Parkinson’s disease (Moller et al., 2005; Styczynska-Soczka et al., 2017). Some studies speculated that Parkinson’s disease may be related to estrogen secretion (Shulman, 2002). Women with higher lifetime estrogen levels have a lower risk of Parkinson’s disease (Gatto et al., 2014). However, the relationship between these metabolites and the sow heat return needs to be further confirmed.

The module composed of vitamin D and its derivatives was enriched in normal return sows, and positively correlated with the CAG17, which was also enriched in normal return sows. Several studies have shown that vitamin D, especially 1α, 25-(OH)2-vitamin D3, has a great influence on the reproductive performance of sows (Lauridsen et al., 2010). 1α, 25-(OH)2-vitamin D3 can regulate the calcium and phosphorus metabolism, and enhance calcium and phosphorus absorption, which benefits sows by quickly restoring body condition after weaning and shortening the interval from weaning to estrus (Jie et al., 2018). The metabolites having the higher abundances in normal return sows were also enriched in the pathway of Steriod hormone biosynthesis. As we have known, many steroid hormones, including sex hormones, play a very important role in the reproductive physiology of pigs. Steroid hormones regulate the endocrine balance through the hypothalamic–pituitary–gonadal axis (HPGA) (Hengevoss et al., 2015), thereby affecting the sow’s sexual cycle and estrus. The metabolites enriched in the serum of non-return sows have been reported to inhibit estrus expressions, such as tropane alkaloids, indole and its derivatives, sulfoxides, lignans, and neolignans. Atropine belongs to tropane alkaloids that can inhibit the effect of oxytocin in goats during estrus (Fodor and Dharanipragada, 1994). Indoles have anti-gonadotropin effects (Reiter and Vaughan, 1977). All these metabolites were correlated with heat return-associated gut microbiota. We speculate that the gut microbiota can participate in the physiological processes of estrus in sows through another manner by regulating the metabolites related to the steriod hormone biosynthesis or influencing the effect of sex hormone. However, the causality and the underlying mechanisms have not been elucidated. Further studies are needed to investigate the mechanism of the key microbiota and metabolites identified in this study affecting estrus return of sows after weaning.

In summary, this study innovatively investigated the effect of sow gut and vaginal microbiota on non-return of sows after weaning and found several microbes that may affect the return of sow estrus. The metabolites and metabolic modules show different abundances between normal return and non-return sows and may be used as markers associated with the return of sow estrus after weaning. The correlation analysis between the gut microbiota and serum metabolites indicated that the changes of gut microbiome should be related to the shifts of serum metabolites between normal return and non-return sows. These results provide new insights into the understanding of the effect of gut and vaginal microbiota on sow estrus return after weaning and suggest that gut and vaginal microbiota may be treated as a target regulating sow estrus. Unfortunately, only 13 serum samples were collected and the metabolomics analysis was performed. In a future study, we will collect more serum samples for metabolomics analysis to further confirm the relationship of fecal microbiota and serum metabolites and elucidate the possible mechanism of the gut and vagina microbes affecting sow estrus return by integrating the multi-omics data.

## Data Availability Statement

The datasets generated from this study were submitted to China National GeneBank Database (CNGBdb) with accession code: CNP0001739 .

## Ethics Statement

The animal study was reviewed and approved by the Ministry of Agriculture and Rural Affairs of China. This study was approved by the Animal Care and Use Committee (ACUC) at Jiangxi Agricultural University (No. JXAU2011-006).

## Author Contributions

LH designed this study and revised the manuscript. CC designed the experiments and wrote and revised the manuscript. JZ analyzed the data and wrote the manuscript. ML analyzed the data and revised the manuscript. SK, XH, SF, MH, and HF performed the experiments. All authors read and approved the final manuscript.

## Conflict of Interest

The authors declare that the research was conducted in the absence of any commercial or financial relationships that could be construed as a potential conflict of interest.

## Publisher’s Note

All claims expressed in this article are solely those of the authors and do not necessarily represent those of their affiliated organizations, or those of the publisher, the editors and the reviewers. Any product that may be evaluated in this article, or claim that may be made by its manufacturer, is not guaranteed or endorsed by the publisher.

## Supplementary Material

Supplementary Figure 1 The rarefaction curve of 16S rRNA gene sequencing data. **(A)** Fecal samples. **(B)** Vaginal samples.

Supplementary Figure 2 Comparison of α-diversity and β-diversity of gut microbiota between normal return and non-return Sows. **(A)** Comparison of the α-diversity (observed species and Chao index) in the fecal samples between normal return and non-return sows. Wilcoxon rank-sum test was used for comparison analysis. **(B)** PCoA of gut microbiota based on Weighted UniFrac distance and Unweighted UniFrac distance between the normal return and non-return sows. PERMANOVA was used for significant test. The number of permutations was set at 10000 times. *p* < 0.05 means achievement of significant level, *R*^2^ value suggested the degree of interpretation of the differences in the sample by the grouping factors.

Supplementary Figure 3 Comparative analysis of the α-diversity and β-diversity of microbiota between normal return and non-return sows in vaginal swab samples. **(A)** Comparison of the α-diversity (observed species and Chao index) in the vaginal samples between normal return and non-return sows. **(B)** PCoA of vaginal microbiota based on Weighted UniFrac distance and Unweighted UniFrac distance between the normal return and non-return sows.

Supplementary Figure 4 The OTUs showing different abundances between normal return and return and non-return sows in 13 feces samples from sows that had serum metabolome data. LEfSe analysis was used to identify the differential OTUs between the two groups (| LDA| score > 2, *p* < 0.05).

Supplementary Figure 5 Comparison of the microbial compositions of feces and vaginal swab samples among seven parities by PCoA based on weighted UniFrac distance. **(A)** Feces samples; **(B)** vaginal swab samples. The results showed that the effect of parity on the microbial compositions of feces and vaginal swab samples was not significant. The effect size (*R*^2^) was estimated via envfit (vegan) based on weighted UniFrac distance.

Supplementary Table 1 The OTUs showing differential abundances between normal return and non-return sows in 158 fecal samples.

Supplementary Table 2 The OTUs are clustered into co-abundance groups (CAGs) by the SparCC Algorithm.

Supplementary Table 3 Differential KEGG pathways of fecal and vaginal microbiome between normal return and non-return sows.

Supplementary Table 4 Differential serum metabolites between normal return and non-return sows.

Supplementary Table 5 Differential metabolite modules of serum compounds via WGCNA method between normal return and non-return sows.
