# Supplementary figures and images for: Gut and Vagina Microbiota Associated With Estrus Return of Weaning Sows and Its Correlation With the Changes in Serum Metabolites
Source: Front Microbiol. 2021 Aug 19;12:690091. doi: 10.3389/fmicb.2021.690091 (PMC8417050; doi:10.3389/fmicb.2021.690091)

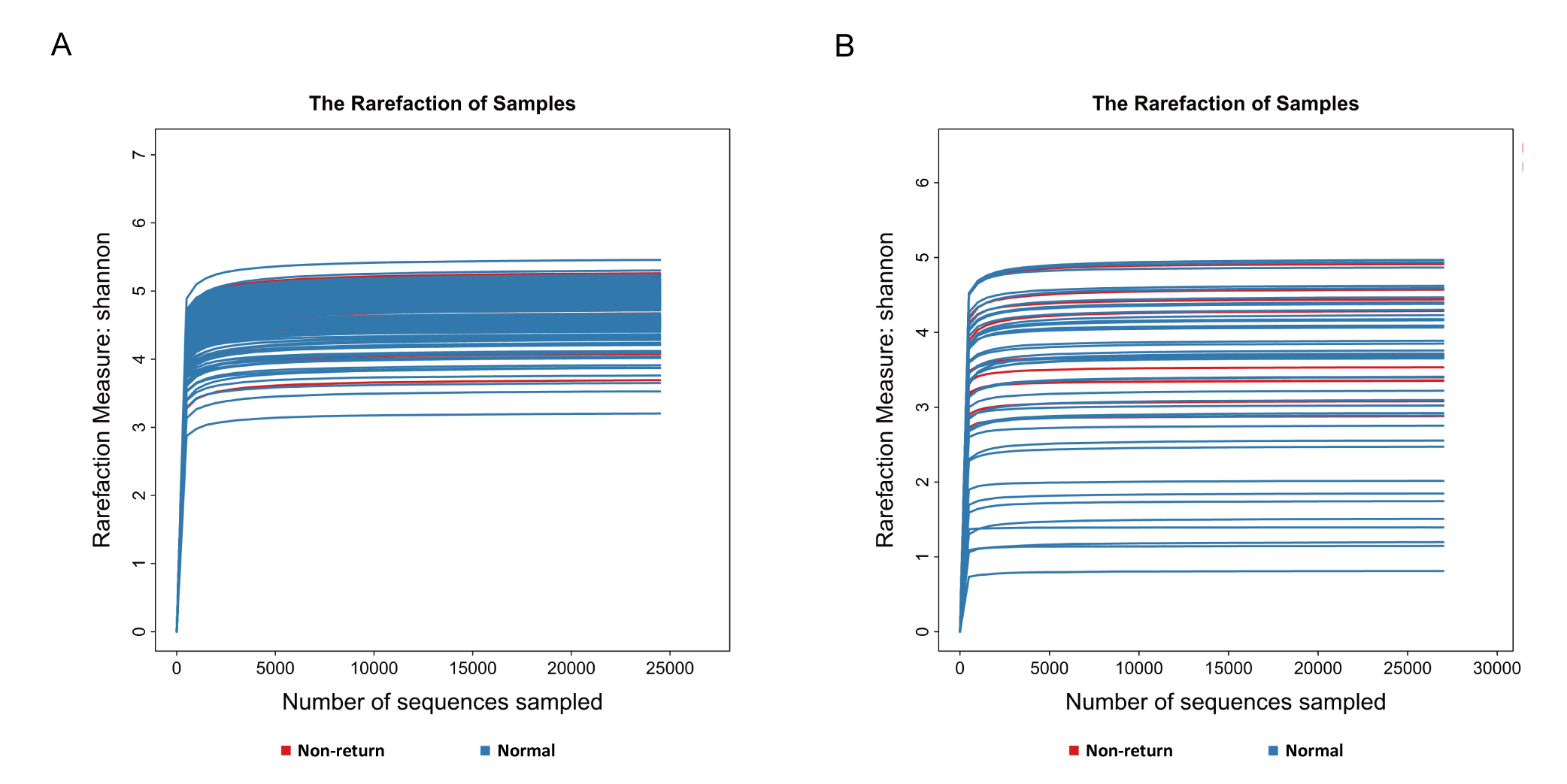

Supplement: Supplementary Figure 1 — The rarefaction curve of 16S rRNA gene sequencing data. (A) Fecal samples. (B) Vaginal samples. [file Image_1.TIF]

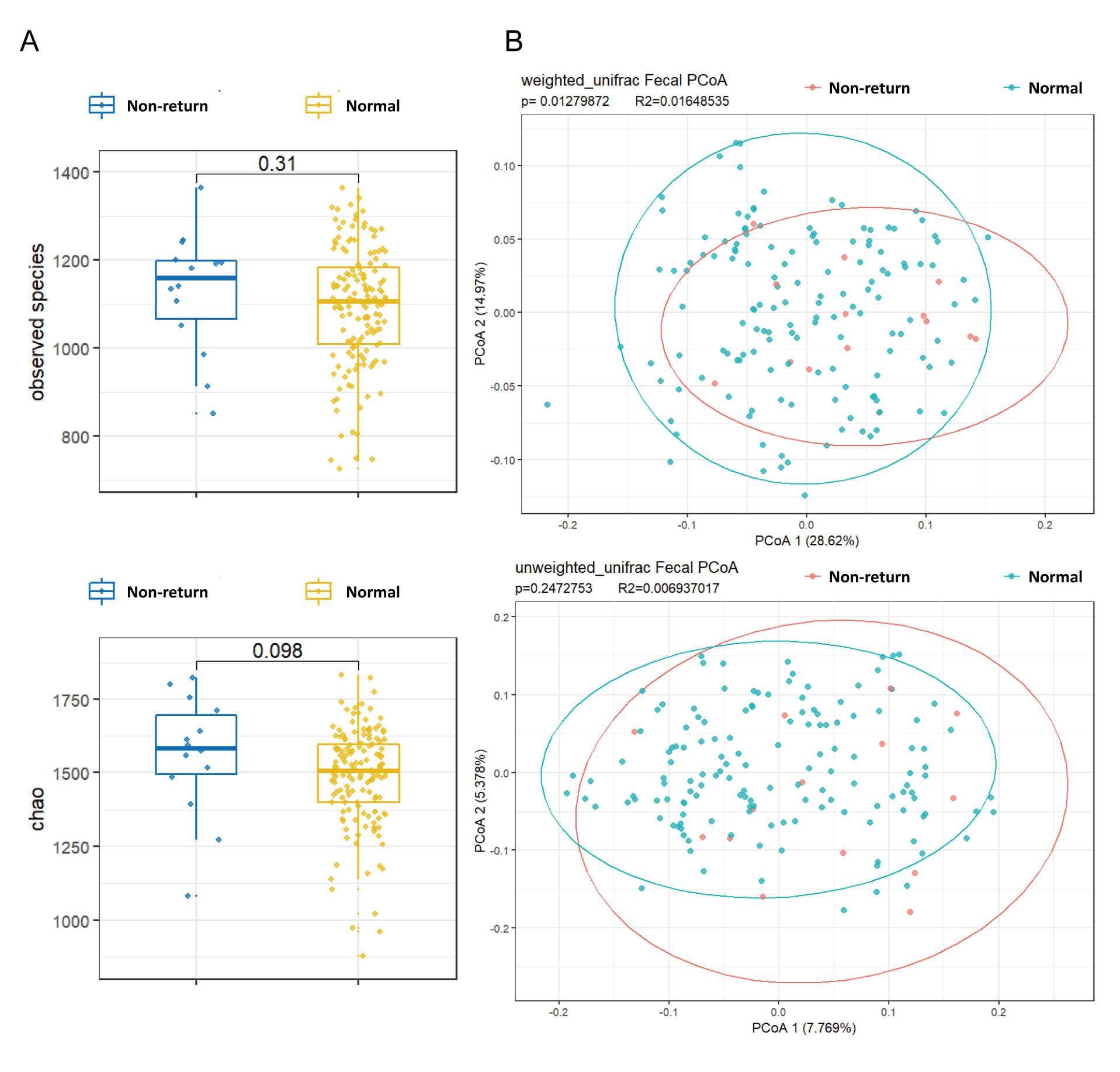

Supplement: Supplementary Figure 2 — Comparison of α-diversity and β-diversity of gut microbiota between normal return and non-return Sows. (A) Comparison of the α-diversity (observed species and Chao index) in the fecal samples between normal return and non-return sows. Wilcoxon rank-sum test was used for comparison analysis. (B) PCoA of gut microbiota based on Weighted UniFrac distance and Unweighted UniFrac distance between the normal return and non-return sows. PERMANOVA was used for significant test. The number of permutations was set at 10000 times. p < 0.05 means achievement of significant level, R2 value suggested the degree of interpretation of the differences in the sample by the grouping factors. [file Image_2.TIF]

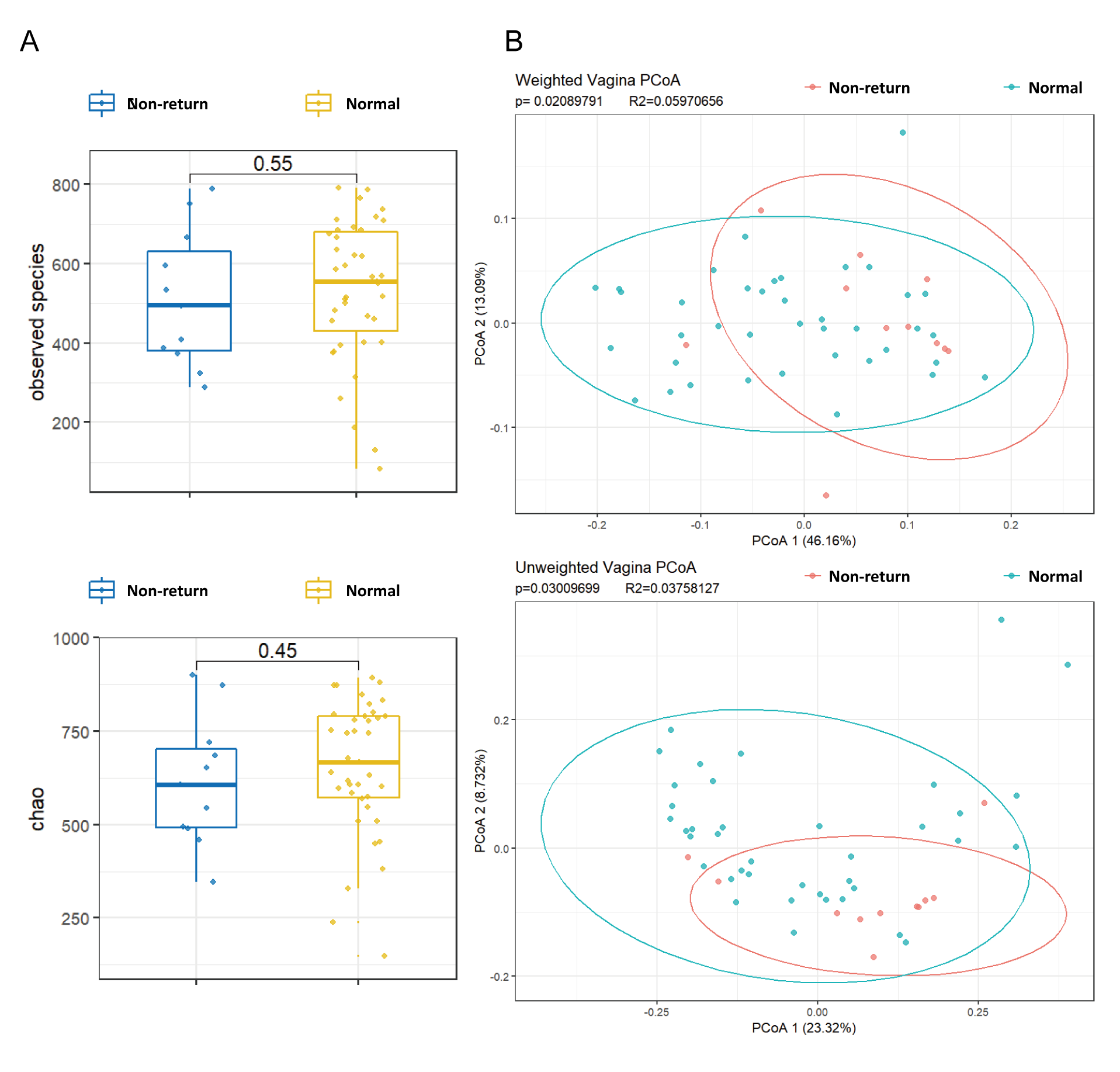

Supplement: Supplementary Figure 3 — Comparative analysis of the α-diversity and β-diversity of microbiota between normal return and non-return sows in vaginal swab samples. (A) Comparison of the α-diversity (observed species and Chao index) in the vaginal samples between normal return and non-return sows. (B) PCoA of vaginal microbiota based on Weighted UniFrac distance and Unweighted UniFrac distance between the normal return and non-return sows. [file Image_3.TIF]

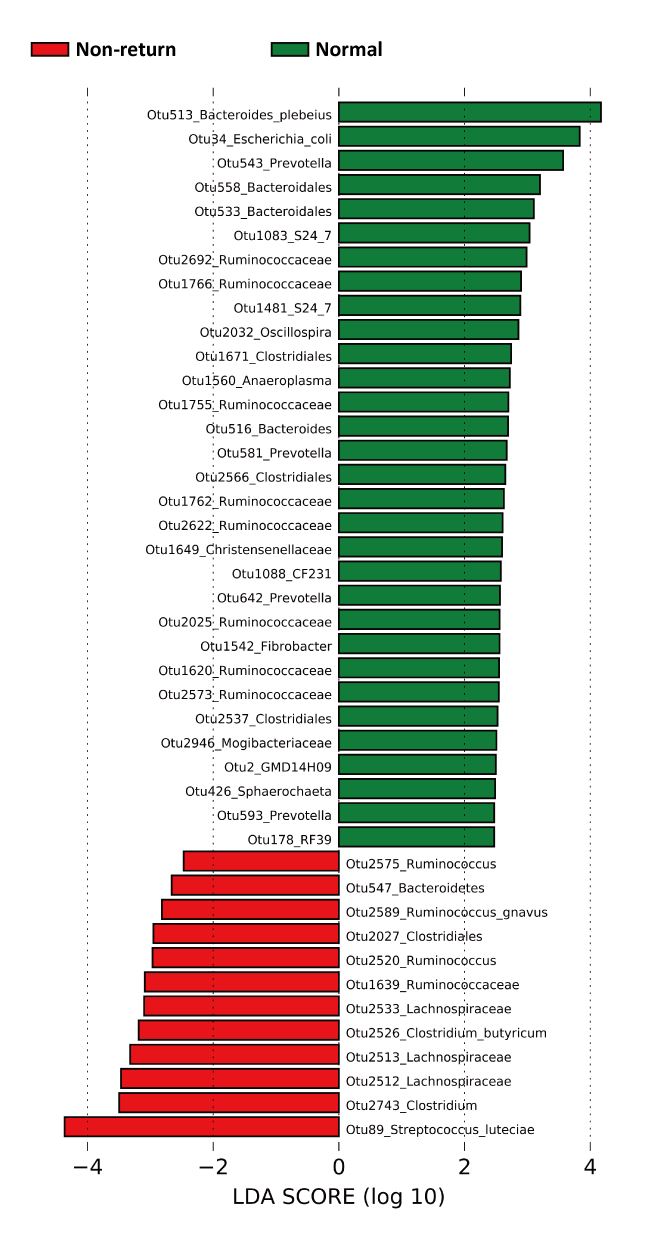

Supplement: Supplementary Figure 4 — The OTUs showing different abundances between normal return and return and non-return sows in 13 feces samples from sows that had serum metabolome data. LEfSe analysis was used to identify the differential OTUs between the two groups (| LDA| score > 2, p < 0.05). [file Image_4.TIF]

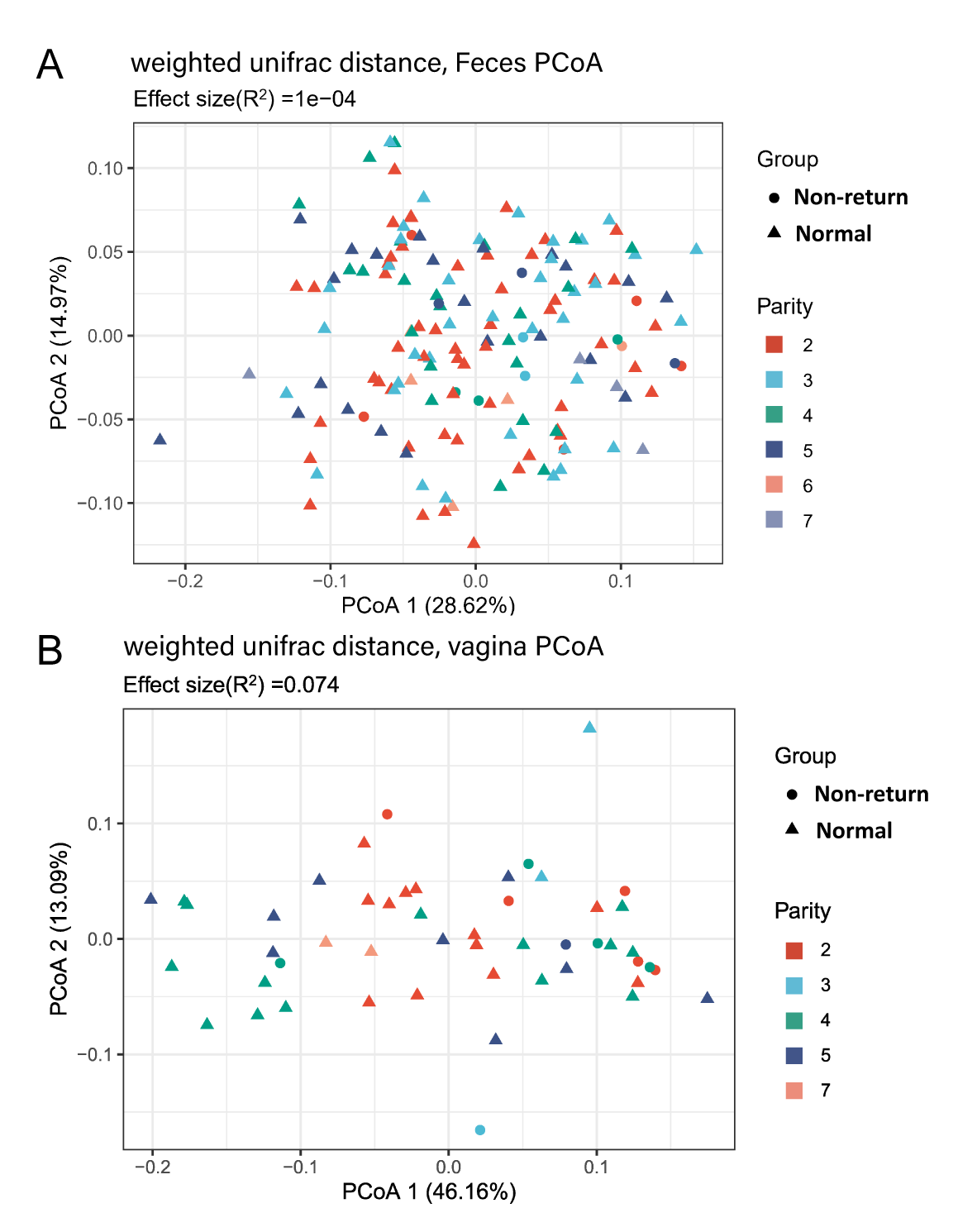

Supplement: Supplementary Figure 5 — Comparison of the microbial compositions of feces and vaginal swab samples among seven parities by PCoA based on weighted UniFrac distance. (A) Feces samples; (B) vaginal swab samples. The results showed that the effect of parity on the microbial compositions of feces and vaginal swab samples was not significant. The effect size (R2) was estimated via envfit (vegan) based on weighted UniFrac distance. [file Image_5.TIF]
